# Supplementary material for: Design of Peptoid-peptide Macrocycles to Inhibit the β-catenin TCF Interaction in Prostate Cancer
Source: Nat Commun. 2018 Oct 23;9:4396. doi: 10.1038/s41467-018-06845-3 (PMC6199279; doi:10.1038/s41467-018-06845-3)
Supplement: Supplementary file 1 — Supplementary Information [file 41467_2018_6845_MOESM1_ESM.pdf]

Supplementary Information

Design of Peptoid-peptide Macrocycles to Inhibit the β-catenin TCF Interaction in Prostate Cancer

Schneider and Craven et al.

Supplementary Figures

a

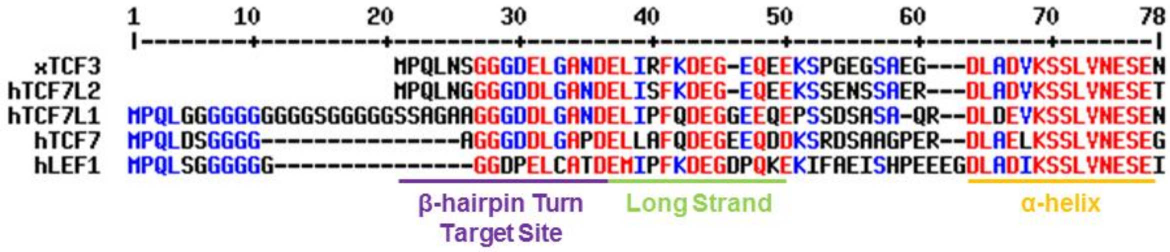

b

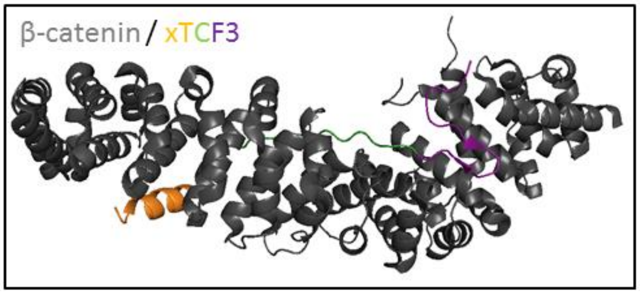

c

Common Name TCF = "T-cell factor"	Gene Name TCF = "Transcription Factor"
TCF1	TCF7
TCF3	TCF7L1
TCF4	TCF7L2
LEF1	LEF1

Supplementary Figure 1. Catenin binding domains of TCF/LEF family members. **(a)** Alignment of the amino acid sequence for the catenin binding domains for human TCF/LEF family members with xenopus TCF3. **(b)** Crystal structure of xTCF3 bound to β-catenin, xTCF3 colors denote each region as marked in panel a. **(c)** Table for comparing common TCF/LEF T-Cell Factor names to Transcription Factor gene names.

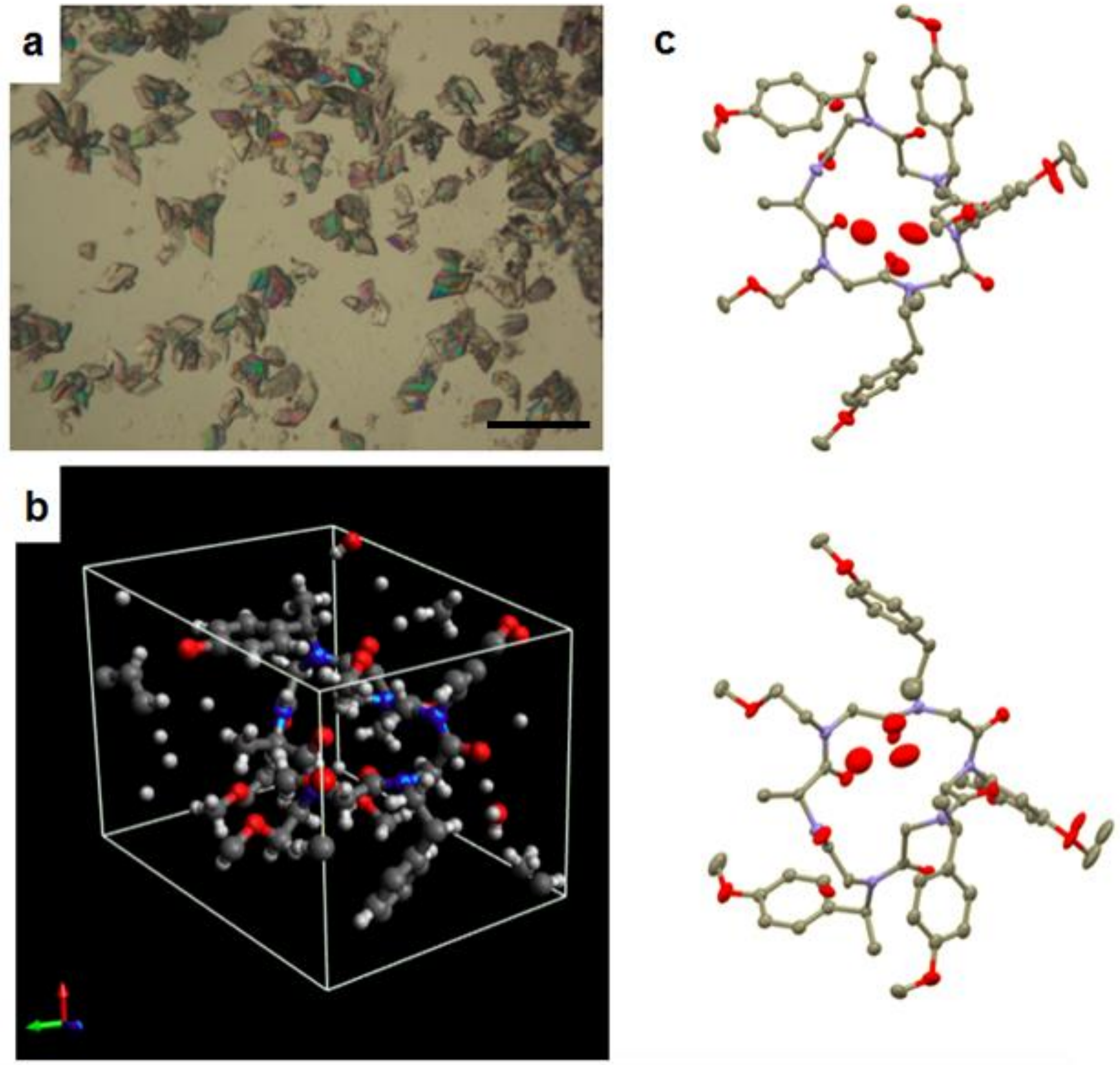

Supplementary Figure 2. Crystallization and X-ray structure determination of compound **1**. **(a)** Crystals of **1** obtained after ~7 days upon slow evaporation of 1 mL of a 50:50 solution of water and methanol and ~5 mg of **1**. Scale bar represents 400 μm. **(b)** Unit cell depiction of the X-ray structure of **1**. **(c)** Ellipsoid representation of the heavy atoms in **1**. **Note:** Unit cell contains 2 water molecules and 1 methanol molecule from crystallization solvent.

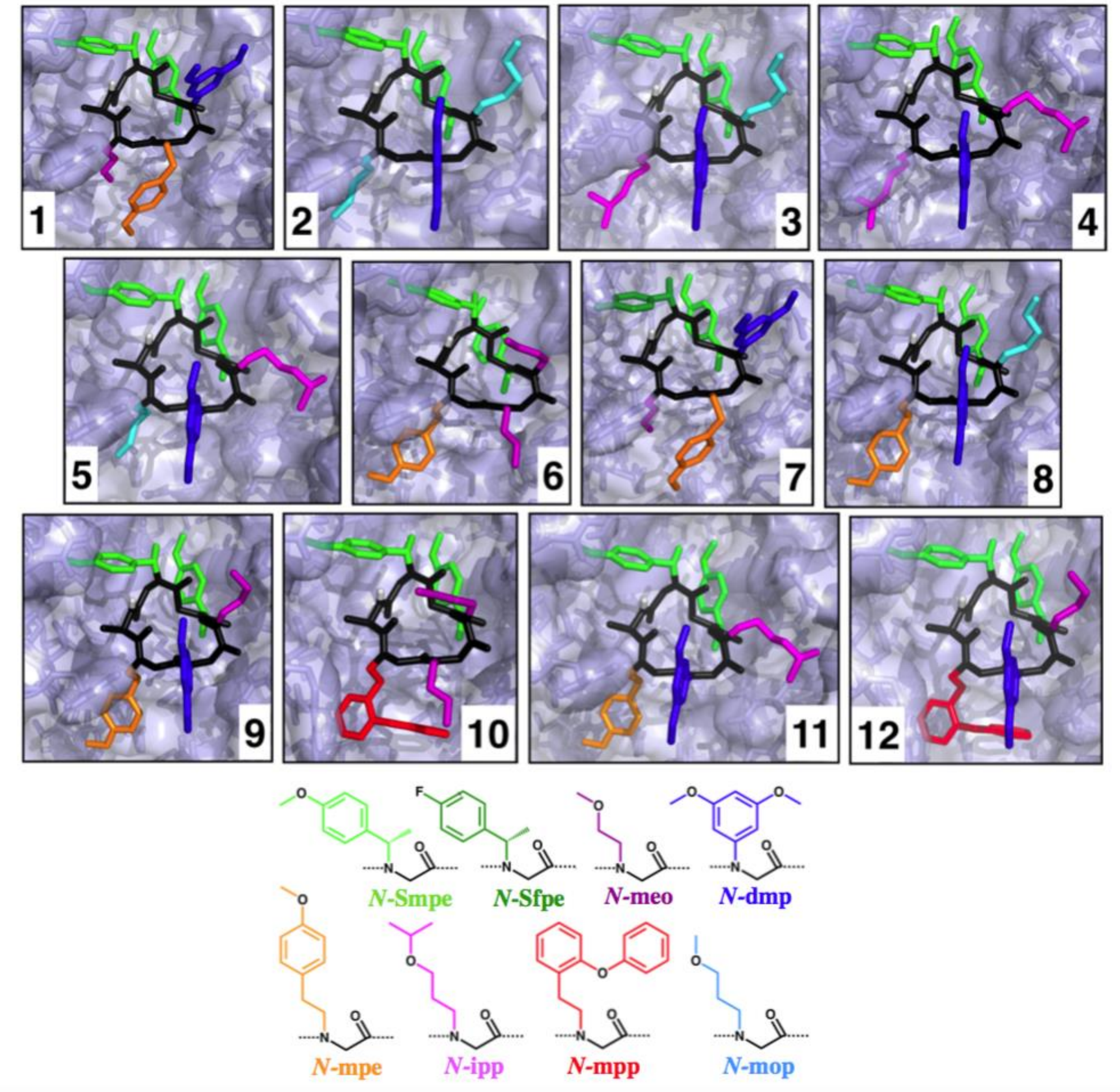

Supplementary Figure 3. Modeling of macrocycles on the surface of β-catenin. Depictions of the lowest energy docked conformations of **Compounds 1-12** found in Figure 2 in the N-terminal TCF binding region of β-catenin (in light blue).

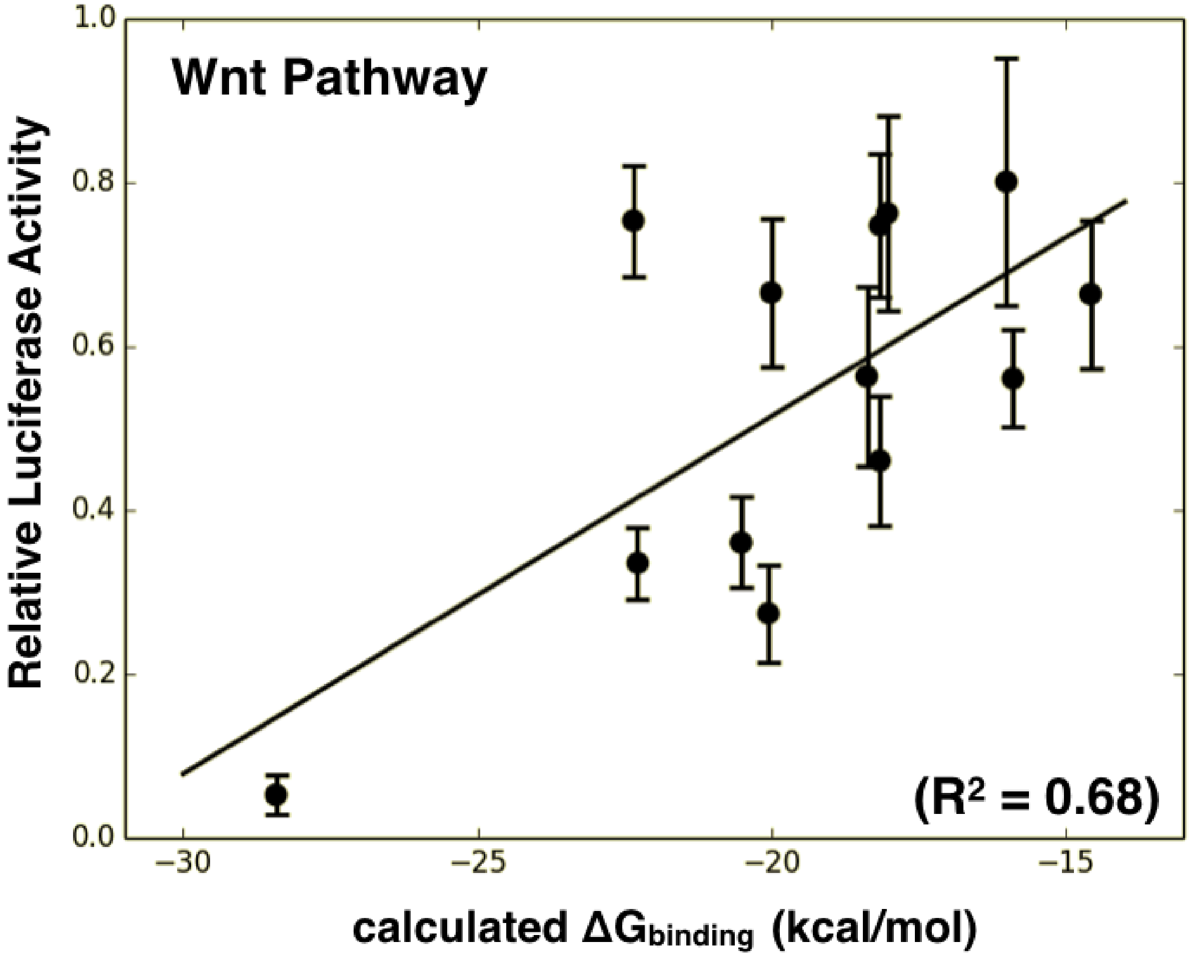

Supplementary Figure 4. Wnt inhibition vs. the calculated binding energies. Relationship of the calculated $\Delta G_{binding}$ for **Compounds 1-13** (values can be found in Supplementary Table 1) versus the observed relative luciferase activity at 10 μM macrocycle concentrations in the Wnt signaling reporter assay. Data are presented as mean and SD (n=3).

a

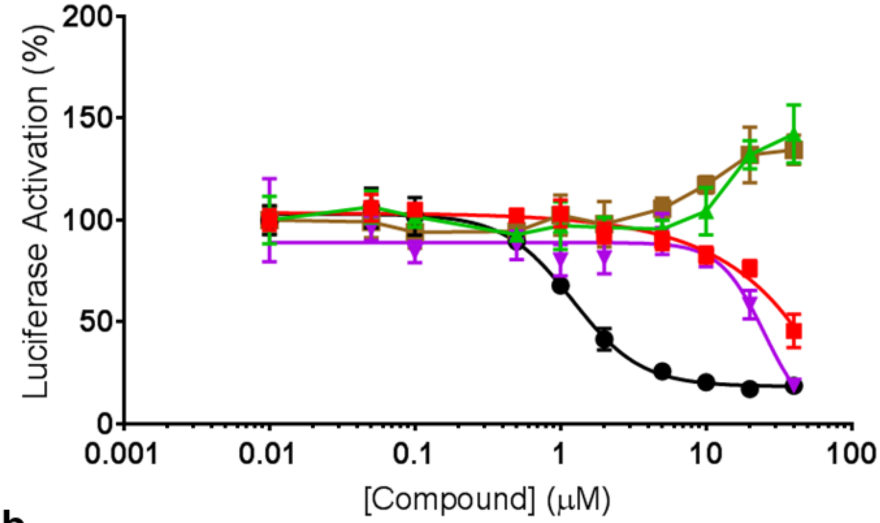

b

Compound	Target	IC50 (μM)	Supplemental Reference
iCRT3	β-catenin/TCF	1.21 +/- 0.07	1
LF3	β-catenin/TCF	>40	2
PRI-724	β-catenin/CBP	25.1 +/- 9.6	3
IWR-1	Tankyrase	N/A	4
IWP-2	Porcupine	N/A	4

Supplementary Figure 5. Control Wnt inhibitors activity in Wnt luciferase assay. **(a)** Dose response curves of 5 different previously developed Wnt inhibitors in the Wnt luciferase assay. **(b)** Color code, target, IC50, and supplemental references for each Wnt inhibitor[1-4]. Data are presented as mean and SD (n=4) of a representative of two independent experiments.

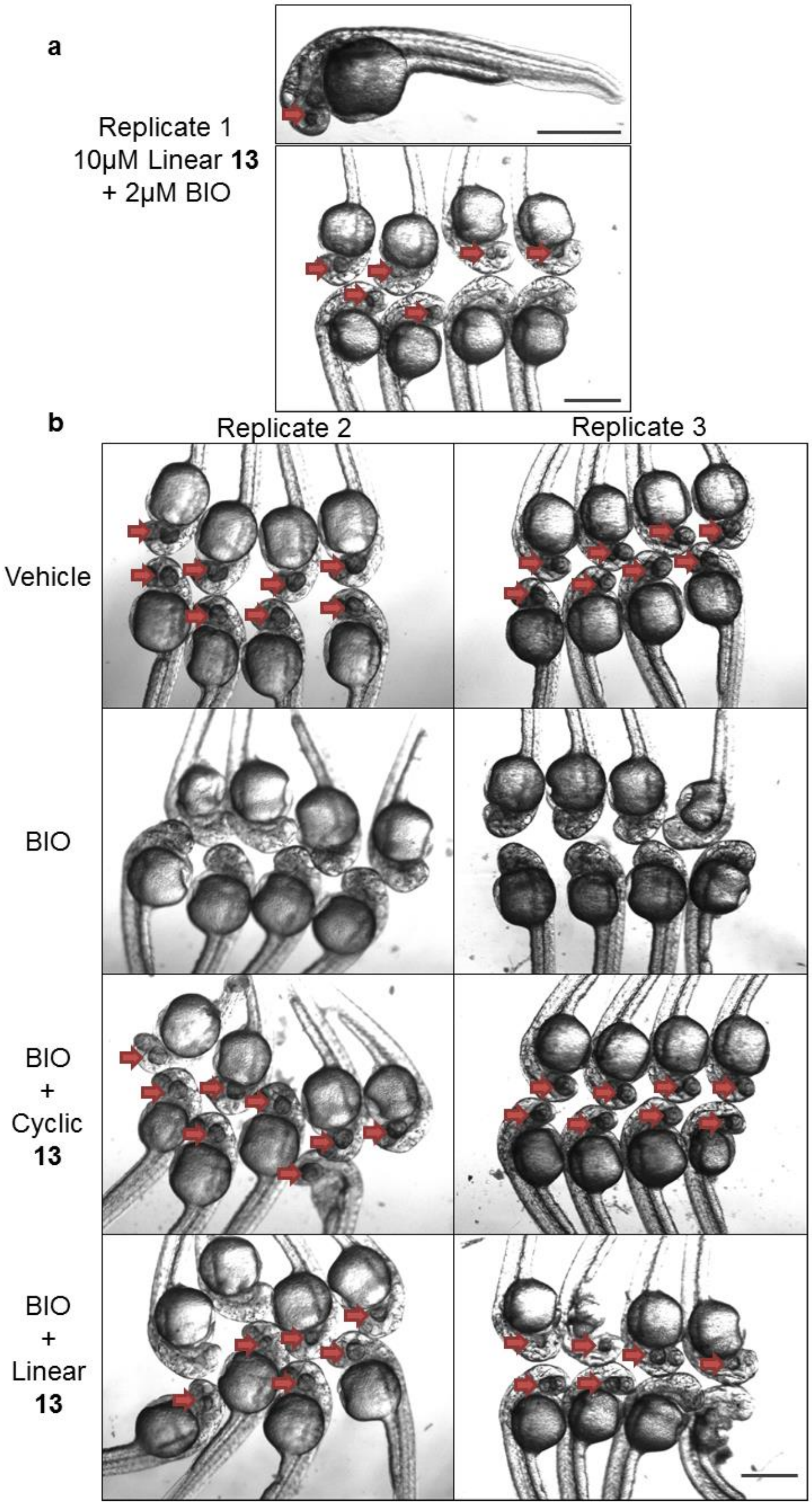

Supplementary Figure 6. Zebrafish experiment replicates. **(a)** Zebrafish treated with 10 μM Linear **13** and 2μM BIO corresponding to Fig. 6. **(b)** Replicates for zebrafish experiments. Red arrows point to developing eye structures. Scale bars represent 500 μm.

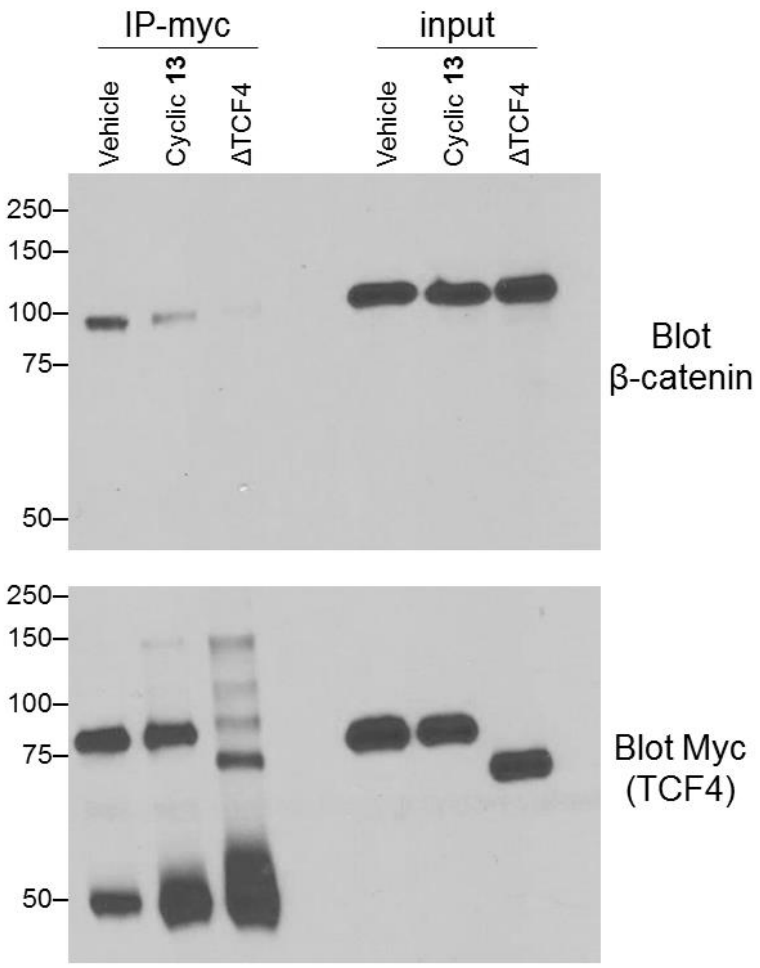

Supplemental Figure 7. Full western blots from immunoprecipitation experiment.

Supplementary Tables

Supplementary Table 1. Unit cell parameters for crystals of **1** used for X-ray determination.

**Space Group**	**P1**		
**Cell Lengths**	**a: 11.431(2)**	**b: 11.631(2)**	**c: 12.105(3)**
**Cell Angles**	**α: 114.596(3)**	**β: 111.002(3)**	**γ: 96.789(4)**
**Cell Volume**	**1296.62**		
**Z, Z'**	**Z: 1 Z':0**		
**R-Factor (%)**	**6.25**		

Supplementary Table 2. Modeling metrics for compounds **1-13**.

	calculated $\Delta G_{binding}$ (kcal/mol)	shape complimentarily	Δ SASA interface (Å²)	Δ SASA polar (Å²)	Δ SASA hydrophobic (Å²)
Compound 1	-15.8982	0.439813	1151.5	414.743	736.754
Compound 2	-16.0074	0.444125	1112.42	332.554	779.864
Compound 3	-18.035	0.52024	1178.67	452.441	726.233
Compound 4	-22.3507	0.526814	1298.32	320.875	977.448
Compound 5	-18.1555	0.468027	1192.2	301.082	891.119
Compound 6	-20.0088	0.446744	1235.37	390.083	845.292
Compound 7	-14.5798	0.396349	1089.45	387.565	701.885
Compound 8	-18.3853	0.391991	1257.71	398.555	859.151
Compound 9	-18.1607	0.412817	1220.19	387.653	832.537
Compound 10	-20.519	0.519971	1288.65	452.346	836.301
Compound 11	-22.2764	0.413352	1338.35	337.868	1000.48
Compound 12	-20.0559	0.507976	1301.96	452.674	849.286
Compound 13	-28.4243	0.539725	1466.47	522.41	944.061

Interface metrics for the lowest energy docked conformations of **Compounds 1-13** presented in Supplementary Fig. 3. Calculated $\Delta G_{binding}$ was found using the InterfaceAnalyzer Protocol in the Rosetta Molecular Design Suite using the 'beta' score function[5,6]. The shape complementarity was calculated using an implementation of the CCP4 algorithm within Rosetta. 'SASA' : solvent-accessible surface area[7].

Supplementary Table 3. Dynamic light scattering of β-catenin.

β-catenin + Vehicle

Replicate 1	Radius (nm)	%Pd	%Mass	Replicate 2	Radius (nm)	%Pd	%Mass	Replicate 3	Radius (nm)	%Pd	%Mass
**Peak 1**	**5.0**	**5.4**	**98.6**	**Peak 1**	**5.0**	**7.1**	**99.3**	**Peak 1**	**5.1**	**9.6**	**100.0**
Peak 2	18.2	11.9	0.5	Peak 2	31.7	7.9	0.1	Peak 2	61.0	11.4	0.0
Peak 3	136.3	0.0	0.9	Peak 3	139.6	7.4	0.6				

β-catenin + Compound 13

Replicate 1	Radius (nm)	%Pd	%Mass	Replicate 2	Radius (nm)	%Pd	%Mass	Replicate 3	Radius (nm)	%Pd	%Mass
**Peak 1**	**5.9**	**9.6**	**30.6**	**Peak 1**	**6.0**	**8.5**	**40.1**	**Peak 1**	**6.2**	**3.2**	**40.9**
Peak 2	222.4	5.9	49.6	Peak 2	25.9	0.0	0.4	Peak 2	71.9	11.2	0.3
Peak 3	661.5	11.2	19.8	Peak 3	237.4	11.4	59.5	Peak 3	286.6	8.3	58.8

β-catenin + α-cyclodextrin

Replicate 1	Radius (nm)	%Pd	%Mass	Replicate 2	Radius (nm)	%Pd	%Mass	Replicate 3	Radius (nm)	%Pd	%Mass
**Peak 1**	**5.1**	**9.0**	**82.2**	**Peak 1**	**4.8**	**5.2**	**100.0**	**Peak 1**	**4.9**	**3.2**	**100.0**
Peak 2	51.3	7.3	0.1	Peak 2	38.4	11.0	0.0	Peak 2	57.4	11.6	0.0
Peak 3	224.5	7.4	3.2								
Peak 4	5877.6	8.3	14.5								

Raw data from replicates of DLS experiments. %Pd = %Polydispersity. Peak 1 is β-catenin with or without compound **13** or α-cyclodextrin. Peaks 2-4 are considered to be β-catenin aggregates due to large radii values.

Supplementary Table 4. Key for chemical abbreviations.

Abbreviation	Chemical Name
DMF	dimethylformamide
DIC	diisopropylcarbodiimide
DCM	dichloromethane
HFIP	hexafluoroisopropanol
ACN	acetonitrile
PyBop	benzotriazol-1-yl-oxytripyrrolidinophosphonium hexafluorophosphate
DIEA	N,N-Diisopropylethylamine

Supplementary table 5. Primers used for RT-qPCR.

Gene	Forward Primer	Reverse Primer
**RPL19**	CACAAGCTGAAGGCAGACAA	GCGTGCTTCCTTGGTCTTAG
**AR**	TACCAGCTCACCAAGCTCCT	GAACTGATGCAGCTCTCTCG
**AR-V7**	CCATCTTGTCGTCTTCGGAAATGTTA	TTTGAATGAGGCAAGTCAGCCTTTCT
**Myc**	TCGGAAGGACTATCCTGCTG	GTGTGTTCGCCTCTTGACATT
**CycD1**	CCGTCCATGCGGAAGATC	GAAGACCTCCTCCTCGCACT
**E-Cad**	TGAAGGTGACAGAGCCTCTGGAT	TGGGTGAATTCGGGCTTGTT
**PSA**	CCAAGTTCATGCTGTGTGCT	GCACACCATTACAGACAAGTGG
**FKBP5**	CGCAGGATATACGCCAACAT	CTTGCCCATTGCTTTATTGG

All primers are shown starting at the 5’ end.

Supplementary References

1. Gonsalves, F.C. et al. An RNAi-based chemical genetic screen identifies three small-molecule inhibitors of the Wnt/wingless signaling pathway. *Proc Natl Acad Sci U S A* **108**, 5954-63 (2011).
2. Fang, L. et al. A Small-Molecule Antagonist of the beta-Catenin/TCF4 Interaction Blocks the Self-Renewal of Cancer Stem Cells and Suppresses Tumorigenesis. *Cancer Res* **76**, 891-901 (2016).
3. Emami, K.H. et al. A small molecule inhibitor of beta-catenin/CREB-binding protein transcription [corrected]. *Proc Natl Acad Sci U S A* **101**, 12682-7 (2004).
4. Chen, B. et al. Small molecule-mediated disruption of Wnt-dependent signaling in tissue regeneration and cancer. *Nat Chem Biol* **5**, 100-7 (2009).
5. Stranges, P.B. & Kuhlman, B. A comparison of successful and failed protein interface designs highlights the challenges of designing buried hydrogen bonds. *Protein Sci* **22**, 74-82 (2013).
6. Park, H. et al. Simultaneous Optimization of Biomolecular Energy Functions on Features from Small Molecules and Macromolecules. *J Chem Theory Comput* **12**, 6201-6212 (2016).
7. Lawrence, M.C. & Colman, P.M. Shape complementarity at protein/protein interfaces. *J Mol Biol* **234**, 946-50 (1993).
